# ECG–gated retinal vessel calibre as a novel measure of aberrant pulsatile retinal flow in diabetes mellitus: a cross-sectional study

**DOI:** 10.1007/s40200-024-01439-x

**Published:** 2024-09-13

**Authors:** Anchal Lal, Michael Anthony (Tony) Barry, Paul Mitchell, Aravinda Thiagalingam

**Affiliations:** 1 Department of Cardiology, Westmead Hospital, Cnr Darcy and Hawkesbury Roads, Sydney, NSW 2145 Australia; 2 Westmead Clinical School, The University of Sydney, Westmead, 2145 Australia; 3 Centre for Vision Research, The Westmead Institute for Medical Research, Sydney, 2145 Australia

**Keywords:** Carotid-femoral pulse wave velocity, Electrocardiogram, Diabetes mellitus, Pulsatile flow, Pulse wave analysis, Retina

## Abstract

**Purpose:**

To evaluate ECG-gated retinal vessel calibre as a novel measure of aberrant pulsatile retinal flow in diabetes mellitus.

**Methods:**

A novel mydriatic ECG-gated fundoscope was used to acquire retinal vessel calibre measurements at four cardiac cycle time points from end diastole to early diastole. An inhouse software recorded the exact time when an image was captured to corroborate ECG-synchronisation. Arterial applanation tonometry, an alternative method of assessing aberrant blood flow, was used to measure carotid-femoral pulse wave velocity (cPWV) and augmentation index (AIx). The intraclass correlation (ICC) was used to perform intra- and inter-observer reliability analyses. Two reviewers measured the retinal vessel calibre in single retinal arterioles and venules. A receiver operating characteristic curve determined associations with diabetes mellitus.

**Results:**

In this study 119 controls and 120 participants with diabetes mellitus were recruited. Mean peak change in retinal arteriolar calibre from baseline was higher in diabetes mellitus compared with controls (controls: 0.92%, IQR 0.63 vs diabetes mellitus: 2.05%, IQR 1.25, *p*<0.0001). In a subset of 9 controls and 11 participants, the intra-and inter-observer reliability was high (ICC 0.87-0.97) in mean peak changes in retinal vascular responses from baseline. In a subset of 36 controls and 95 participants with diabetes mellitus, diabetes mellitus was more strongly associated with retinal arteriolar pulsatility (AUC 0.85, 95%CI 0.76-0.93) than applanation tonometry (cfPWV AUC 0.72, 95%CI 0.62-0.82 vs AIx AUC 0.56, 95%CI 0.45-0.68).

**Conclusion:**

Higher retinal arteriolar pulsatility appears to be more strongly correlated with diabetes mellitus than arterial applanation tonometry.

## Introduction

Arterial stiffness is an independent cardiovascular risk predictor of mortality in diabetes mellitus [1]. It relates to the physiological and anatomical alterations within the arterial wall, resulting in an impairment of vascular compliance, inappropriate responses to changes in blood pressure [2], and a greater pulsatile flow in end organs such as the retina [3].

The association of arterial stiffness with diabetes mellitus [1] has increased interest in developing non-invasive screening tools to detect arterial stiffness early in the pathogenesis of diabetes mellitus. Arterial applanation tonometry is one method that assesses arterial stiffness through carotid-femoral aortic pulse wave velocity (cfPWV) and augmentation index (AIx). The cfPWV is currently the most reliable and valid non-invasive surrogate for estimating arterial stiffness [4], and is in good agreement with the invasive gold standard of catheterisation [5]. Under rigid conditions for arterial applanation tonometry, cfPWV and AIx demonstrate good intra- and inter-observer reproducibility in subjects with diabetes mellitus [6]. However, these conditions are difficult to create and maintain because of the many limitations of both techniques [7, 8]. An alternative method that may overcome many of these limitations includes the direct assessment of retinal vessel calibre [9].

Although the association of systemic arterial stiffness with retinal vessel calibre has been described previously [10], very little has been investigated in diabetes mellitus. The only study [11] to explore this in diabetes mellitus demonstrated that systemic arterial stiffness measured by cardio-ankle vascular index was correlated with ocular microvascular changes in this population. The present study proposes aberrant retinal vessel pulsatile flow as a measure of arterial stiffness in diabetes mellitus, and has developed a novel method of assessing this using mean peak changes in retinal vascular response from baseline based on individual arteriolar and venular calibre measurements (hereby referred to as arteriolar and venular pulsatility). We aimed to determine the associations of diabetes mellitus with our novel method and compare it with the commonly reported method of arterial applanation tonometry measuring cfPWV and AIx.

## Materials and methods

### Study participants and ethics approval

**Fig. 1:**
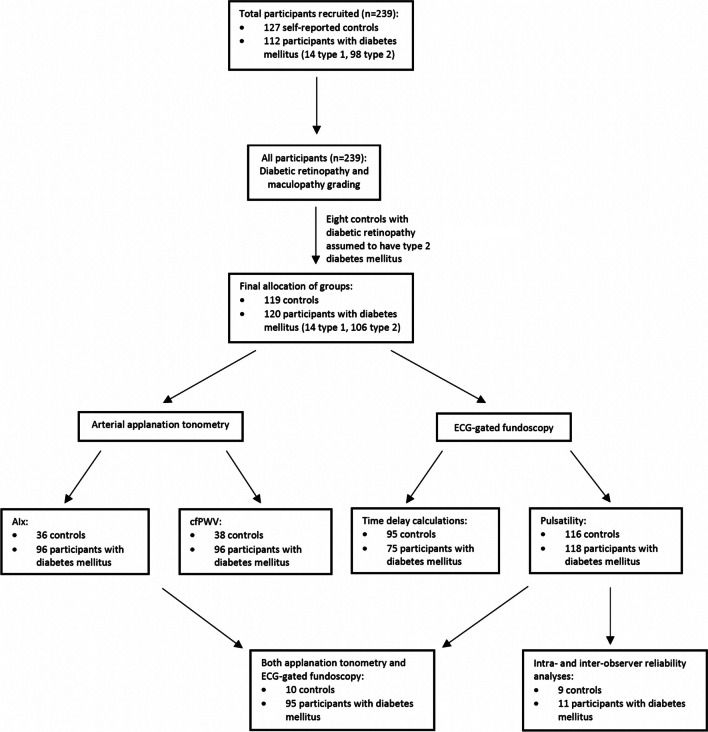
Distribution of controls and participants with diabetes mellitus who completed each component of the Australian Heart and Eye sub-study

A total of 119 controls and 120 participants with diabetes mellitus (14 type 1, 106 type 2) were recruited for this Australian Heart and Eye cross-sectional sub-study at Westmead hospital, Sydney, Australia (Fig. 1). This was achieved through stratified random sampling that prevented selection bias. The study’s total sample size exceeded the original sample size of 50 controls and 50 patients with diabetes mellitus that was calculated to provide 80% power and a 95% level of confidence in detecting a 1% mean difference between the two groups. The study protocol was approved by the Western Sydney Local Health District Human Research Ethics Committee and followed the guidelines of the Declaration of Helsinki. Participants provided informed written consent to partake in this study. Participants were included regardless of pre-existing diabetes-related complications such as diabetic retinopathy. The exclusion criteria included retinal vascular occlusions, glaucoma, and severe cataracts.

### Data collection and anthropometric measurements

Participants were required to fast for 8 hours prior to the commencement of this study including abstaining from food, caffeine, alcohol, medication and smoking, with the exception of water. A detailed history was collected including patient demographics and a past medical history. Fasting glucose was measured in participants with diabetes mellitus and was assumed normal in controls. Anthropometric measurements such as height(m), weight(kg) and waist circumference(cm) were measured. BMI(kg.m^-2^) was calculated by dividing the weight by the height squared. An automated electronic blood pressure device was used to measure the patient’s BP(mmHg) and heart rate(bpm) (Model HEM-907; OMRON Healthcare, Victoria, Australia). MAP(mmHg) was calculated as follows: DBP + 1/3 x pulse pressure. Prior to all examinations, participants were rested for a minimum of 15 minutes.

### Arterial applanation tonometry (SphygmoCor system)

**Fig. 2:**
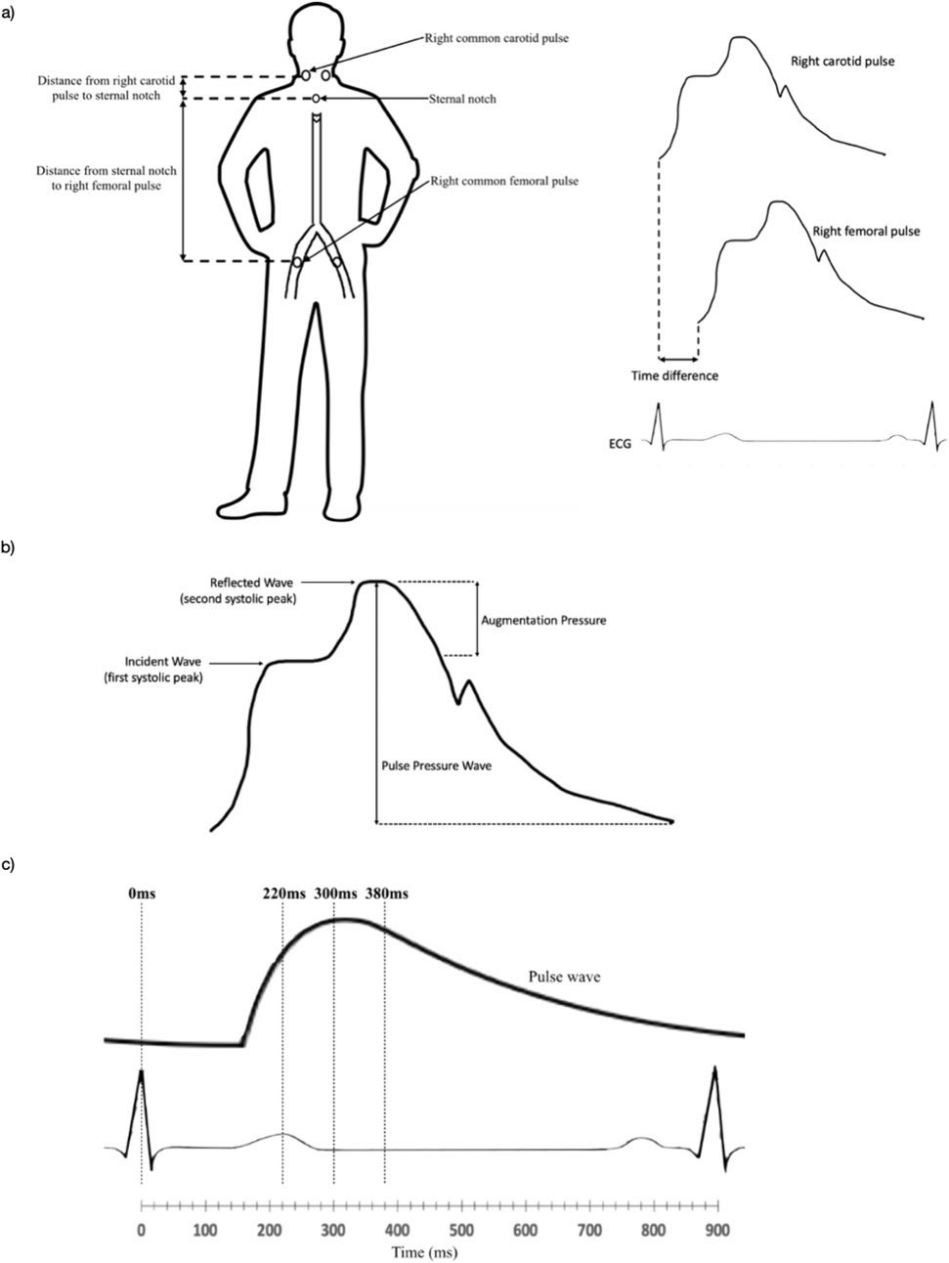
**a**) Visual representation of measuring the distance and the difference in time of pulse wave arrival from the carotid to the femoral site in order to calculate cfPWV **b**) calculation of AIx from the addition of reflected systolic pulse pressure waves **c**) a retinal pulse wave trace during one cardiac cycle with the four time segments of the cardiac cycle where image acquisition was intended to occur. cfPWV, carotid-femoral pulse wave velocity; AIx, Augmentation index

The cfPWV was measured by a widely used commercial system (SphygmoCor, AtCor Medical, West Ryde, Australia) in 38 controls and 96 participants with diabetes mellitus. A three lead ECG in a lead II configuration was used to gate the participant’s R wave as a reference point to quantify the difference in arrival times of the pressure wave at the carotid and femoral pulse points. The difference in arrival time was calculated by subtracting the difference in the foot of the pressure wave at the femoral artery by the difference in the foot of the pressure wave at the carotid artery (Fig. 2). The intersecting tangent method was used to estimate the foot of the waveforms since. Data were captured after 10 consecutive wave forms. The subtraction method was used to estimate the distance of the descending aorta by subtracting the distance of the carotid pulse to the sternal notch from the distance of the sternal notch to the mid-inguinal point. The tape was placed on the right lateral side instead of centrally to avoid over-estimating the distance, especially in obese patients. The cfPWV was calculated as following:

$${\text{cfPWV}}({\text{m}}/{\text{s}})=\frac{\text{Arrival} \,\text{time} \,\text{difference} \,\text{between} \,\text{carotid} \,\text{and} \,\text{femoral} \,\text{pulses}}{\text{Distance} \, \text {between} \,\text{carotid} \,\text{and} \,\text{femoral} \,\text{pulses}}$$

AIx was calculated using radial applanation tonometry (SphygmoCor, AtCor Medical, West Ryde, Australia) by a previously validated software-derived transfer function which estimated central aortic pressure from the peripheral radial pressure. Measurements were performed on the right radial artery in 36 controls and 96 participants with diabetes mellitus. Participants were seated in an upright position with the wrist slightly dorsiflexed and supported by a pillow. The left radial artery was examined if the right radial pulse was difficult to palpate. Radial pressure data were captured after 11 seconds of consistent waveforms with a quality control operator index of ≥80, according to manufacturer’s advice. AIx was defined as the addition of the reflected peripheral pressure wave arriving centrally during systole with the incident systolic pulse pressure wave created from left ventricular ejection (Fig. 2b), and calculated as following:

$$\begin{array}{ll}\text{AIx}(\%)&=\frac{\text{Augmentation}\;\text{pressure}}{\text{Pulse}\;\text{pressure}}\;\times\;100\\&=\frac{\text{Second}\;\text{systolic}\;\text{peak}-\text{first}\;\text{systolic}\;\text{peak}}{\text{Pulse}\;\text{Pressure}}\;\times\;100\end{array}$$

### ECG-gated fundoscope

The Cardiology Research Workshop at our centre devised a retinal ECG adaptor [12] that permitted retinal images to be captured at 0ms, 220ms, 300ms and 380ms after the QRS (Fig. 2c), by gating a mydriatic fundus camera (Canon CF-60DSi; Canon Inc., Tokyo, Japan) to the participant’s ECG. The CF-60DSi was equipped with a 21megapixel full 35mm frame digital camera back (EOS-1Ds Mark III; Canon Inc., Tokyo, Japan). This ECG adaptor reports the smallest time delay in photo acquisition by accounting for the 225ms fixed inherent time delay related to the QRS detection algorithm (120ms) and mechanical parts of the fundus camera (105ms), and is reported in detail elsewhere [12, 13]. A three-lead ECG system was used to acquire the participant’s ECG in real time. An average R-R was established from the participant’s last 60 R-R intervals. The microcontroller permitted image acquisition within a specific range from the participant’s average R-R: -150msec (lower limit) to 125% of the average R-R (upper limit). Sinus arrhythmia, but not ectopic beats, was within this acceptable limit. Image acquisition was possible after 3 consecutive beats within this acceptable range.

### Retinal grading

Digital disc- and macula-centred photographs were graded for diabetic retinopathy and maculopathy according to the Modified Airlie House Classification of DR guidelines [14] and the Wisconsin Age-related Maculopathy Grading System [15] respectively. Digital red-free, optic disc-centred retinal images within a 40-degree field of view were acquired in the left eye for grading retinal vessel calibre. Photographs of the right eye were obtained in 2 individuals with diabetes mellitus since the left eye was unexaminable due to cataracts. Three photographs were captured at each of four time points in the cardiac cycle in 116 controls and 118 participants with diabetes mellitus, including end diastole (0ms after QRS), mid systole (220 ms after QRS), late systole (300 ms after QRS) and early diastole (380 ms after QRS). All images were acquired in RAW format (5632 x 3750 px, 14 bit). An inhouse semi-automated software (RetAligner v1.4.1) calculated the retinal arteriolar and venular calibres within 0.5-1 optic disc diameters from the optic disc edge. The vessel width was estimated at half the peak intensity maximum using the full width half maximum algorithm. Retinal vessel calibre was measured in pixels and multiplied by the scaling factor, which allowed the conversion to microns [16]. The scaling factor was determined by dividing 1800 microns (standard optic disc diameter) [16] by the diameter of the optic disc in pixels. Mean calibres at each of the four intervals of the cardiac cycle were then calculated. Percent changes at 220ms, 300ms, and 380ms after QRS from baseline (QRS) were determined as following:

1. $$\begin{array}{l}\mathrm{Percent}\,\mathrm{change}\,\mathrm{in}\,\mathrm{retinal}\,\mathrm{vessel}\,\mathrm{calibre}\left(220\mathrm{ms}\,\mathrm{after}\,\mathrm{QRS}\right)\mathrm{from}\,\mathrm{baseline}\left(\text{QRS}\right)\\ \;\;\;\;\;\;\;\;\;\;\;\;\;\;=\frac{\mathrm{Retinal}\,\mathrm{vessel}\,\mathrm{calibre}\,\mathrm{at}\,220\mathrm{ms}\,\mathrm{after}\,\mathrm{QRS}\,-\,\mathrm{retinal}\,\mathrm{vessel}\,\mathrm{calibre}\,\mathrm{at}\,\mathrm{QRS}}{\mathrm{Retinal}\,\mathrm{vessel}\,\mathrm{calibre}\,\mathrm{at}\,\mathrm{QRS}}\mathrm x100\end{array}$$  
2. $$\begin{array}{c}\mathrm{Percent}\,\mathrm{change}\,\mathrm{in}\,\mathrm{retinal}\,\mathrm{vessel}\,\mathrm{calibre}\left(300\mathrm{ms}\,\mathrm{after}\,\mathrm{QRS}\right)\mathrm{from}\,\mathrm{baseline}\left(\text{QRS}\right)\\=\frac{\mathrm{Retinal}\,\mathrm{vessel}\,\mathrm{calibre}\,\mathrm{at}\,300\mathrm{ms}\,\mathrm{after}\,\mathrm{QRS}\,-\,\mathrm{retinal}\,\mathrm{vessel}\,\mathrm{calibre}\,\mathrm{at}\,\mathrm{QRS}}{\mathrm{Retinal}\,\mathrm{vessel}\,\mathrm{calibre}\,\mathrm{at}\,\mathrm{QRS}}\mathrm x100\end{array}\begin{array}{c}\\\\\end{array}$$  
3. $$\begin{array}{c}\mathrm{Percent}\,\mathrm{change}\,\mathrm{in}\,\mathrm{retinal}\,\mathrm{vessel}\,\mathrm{calibre}\left(380\mathrm{ms}\,\mathrm{after}\,\mathrm{QRS}\right)\mathrm{from}\,\mathrm{baseline}\left(\text{QRS}\right)\\=\frac{\mathrm{Retinal}\,\mathrm{vessel}\,\mathrm{calibre}\,\mathrm{at}\,380\mathrm{ms}\,\mathrm{after}\,\mathrm{QRS}\,-\,\mathrm{retinal}\,\mathrm{vessel}\,\mathrm{calibre}\,\mathrm{at}\,\mathrm{QRS}}{\mathrm{Retinal}\,\mathrm{vessel}\,\mathrm{calibre}\,\mathrm{at}\,\mathrm{QRS}}\mathrm x100\end{array}$$  

In a subset of 20 participants (9 controls and 10 patients with diabetes mellitus), a second reviewer measured the retinal vessel calibre using randomly selected slice points within the same retinal vessels as the first reviewer.

### Time delay calculations during image acquisition

The timing of image capture was measured at 0ms, 220ms, 300ms and 380ms after the QRS in 95 controls and 75 participants with diabetes mellitus. This was necessary due to variability in the timing of image capture related to factors such as the fundoscope’s mechanical operations and the presence of sinus arrythmia in many people. The latency of the fundoscope between command to acquire an image and the actual image acquisition (as measured by the flash of light into the eye) was constant at 105 msec; but the QRS could be tens of milliseconds away from expected occurrence. Therefore, the actual times were measured using an in-house software (RetECGReporter) which recorded the participant’s ECG trace, identified the time point when the flash button was operated, when the QRS spike occurred, and when flash detection occurred (representing image acquisition). The software tracked and marked the QRS on the ECG trace after the flash using a standard first derivative dV/dT algorithm. A reference point was defined for timing purposes, with the delay at 0ms after the QRS, the reference point being the peak of the first QRS after flash detection. For the delay measurements at 220ms, 300ms and 380ms after the QRS, the reference value was the peak of the last QRS before flash detection. Time delays were measured as the difference between the point of image acquisition and the reference value.

### Statistics

All data were entered onto SPSS for macOs 26.0 (SPSS Inc., Chicago, IL, USA) for statistical analyses. SPSS and Prism version 9.5.0 for macOS (GraphPad Software Inc., San Diego, CA, USA) were used to generate the graphs. Shapiro-Wilk test was used to test the data for normality. Nominal data were organised as mean, standard deviation and 95% confidence intervals, and as median and interquartile ranges where necessary, while categorical data were represented as frequency and percentages. Extreme outliers (below Q_1_ – 3 x IQR and above Q_3_ + 3 x IQR) were excluded when generating and statistically analysing box plots. Pearson’s *χ*^2^ test compared categorical data between groups. In groups with multiple comparisons, a significant *χ*^*2*^ test was followed by Bonferroni *χ*^2^ residual analysis to identify significant pairs between groups.

Independent Mann-Whitney U test compared the median of nominal variables between groups. Paired samples t-tests compared nominal variables within groups and the independent student’s t-test between groups. Spearman’s rho correlation coefficient was used to assess statistically significant strength of associations. The multiple linear stepwise regression model was used to investigate parameters associated with cfPWV, AIx, arteriolar pulsatility and venular pulsatility. A receiver operating characteristic curve determined the association of diabetes mellitus with cfPWV, AIx, arteriolar pulsatility and venular pulsatility. The intraclass correlation coefficient (ICC) was used to perform intra- and inter-observer reliability analyses on 9 controls and 11 participants with diabetes mellitus, using a two-way mixed model with absolute agreement. Statistical significance was attributed at a two-sided *p* value of less than 0.05 for all statistical analyses, unless otherwise indicated by the Bonferroni corrected *p* value.

## Results

Table 1 summarises the participant characteristics of the study. Compared with controls, participants with diabetes mellitus had a higher mean age, body mass index, waist circumference, MAP, SBP, DBP, and heart rate. Caucasians and South Asians formed a large proportion of participants. A greater proportion of participants with diabetes mellitus were ex-smokers and a lower proportion had never smoked, compared with controls. A higher proportion of participants with diabetes mellitus had hypertension, hypercholesterolaemia and fatty liver compared to controls. All participants’ health conditions were well controlled with medications. All participants had no clinically significant age-related maculopathy and the majority of participants had no diabetic retinopathy.

**Table 1:** Participant baseline characteristics

	Controls (n = 119)		DM (n = 120)		
Variable	n (%)	Mean (±SD)	n (%)	Mean (±SD)	*p*-value
Age (years)	110	40.1±12.5	118	47.6±13.1	<0.0001
*Ethnicity*^*a*^	119		120		0.003**
Caucasian	32 (26.9)		38 (31.7)		0.42
South Asian	40 (33.6)		30 (25.0)		0.13
South East Asian	13 (10.9)		5 (4.2)		0.046*
Middle Easterner	9 (7.6)		20 (16.7)		0.03*
Mediterranean	15 (12.6)		4 (3.3)		0.009**
Pacific Islander	3 (2.5)		7 (5.8)		0.19
Mixed race	3 (2.5)		8 (6.7)		0.13
Other	4 (3.4)		8 (6.7)		0.23
Body mass index (kg.m^-2^)	107	25.1±5.8	118	30.3±6.9	<0.0001
Waist circumference (cm)	100	84.9±14.7	113	106.0±21.6	<0.0001
*Sex*	119		120		0.17
Male	49 (41.2)		60 (50.0)		
Female	70 (58.8)		60 (50.0)		
*Blood pressure (mmHg)*	105		118		
Mean arterial pressure		90.9±9.1		96.1±10.0	<0.0001
Systolic blood pressure		119.3±13.8		127.6±16.9	<0.0001
Diastolic blood pressure		76.8±7.8		80.3±8.2	0.001**
Heart Rate (bpm)	106	69.0±11.1	118	78.8±13.8	<0.0001
Fasting glucose	-	-	59	8.6±3.2	-
*Medical condition*					
Diabetes (type1/type 2)	-	-	14/106 (11.7/88.3)	-	-
Diabetes duration (years)	-	-	106	9.5±8.7	-
Hypertension	16 (13.6)		49 (42.6)		<0.0001
Hypercholesterolaemia	17 (14.5)		61 (52.1)		<0.0001
Fatty liver	2 (1.7)		12 (10.0)		0.003**
*Retinopathy grading*^*b*^	119		120		<0.0001
Nil diabetic retinopathy	119 (96.6)		75 (52.5)		<0.0001
Questionable diabetic retinopathy	0 (0.0)		5 (4.2)		0.02*
Minimal-mild NPDR	0 (0.0)		22 (18.3)		<0.0001
Moderate-severe NPDR	0 (0.0)		15 (12.5)		<0.0001
Inactive PDR	0 (0.0)		3 (2.5)		0.09
*Smoking history*^*c*^	119		120		0.0006***
Never smoker	95 (79.8)		69 (57.5)		0.0001***
Current smoker	3 (2.5)		3 (2.5)		1.00
Ex-smoker	21 (17.6)		48 (40.0)		0.0002***

^a^Bonferroni adjusted *p*<0.003

^b^Bonferroni adjusted *p*<0.005

^c^Bonferroni adjusted *p*<0.004

^*^p<0.05, **p<0.01, ***p<0.001

Abbreviations: DM, Diabetes Mellitus; NPDR, Non-Proliferative Diabetic Retinopathy; PDR, Proliferative Diabetic Retinopathy; SD, Standard Deviation

**Fig. 3:**
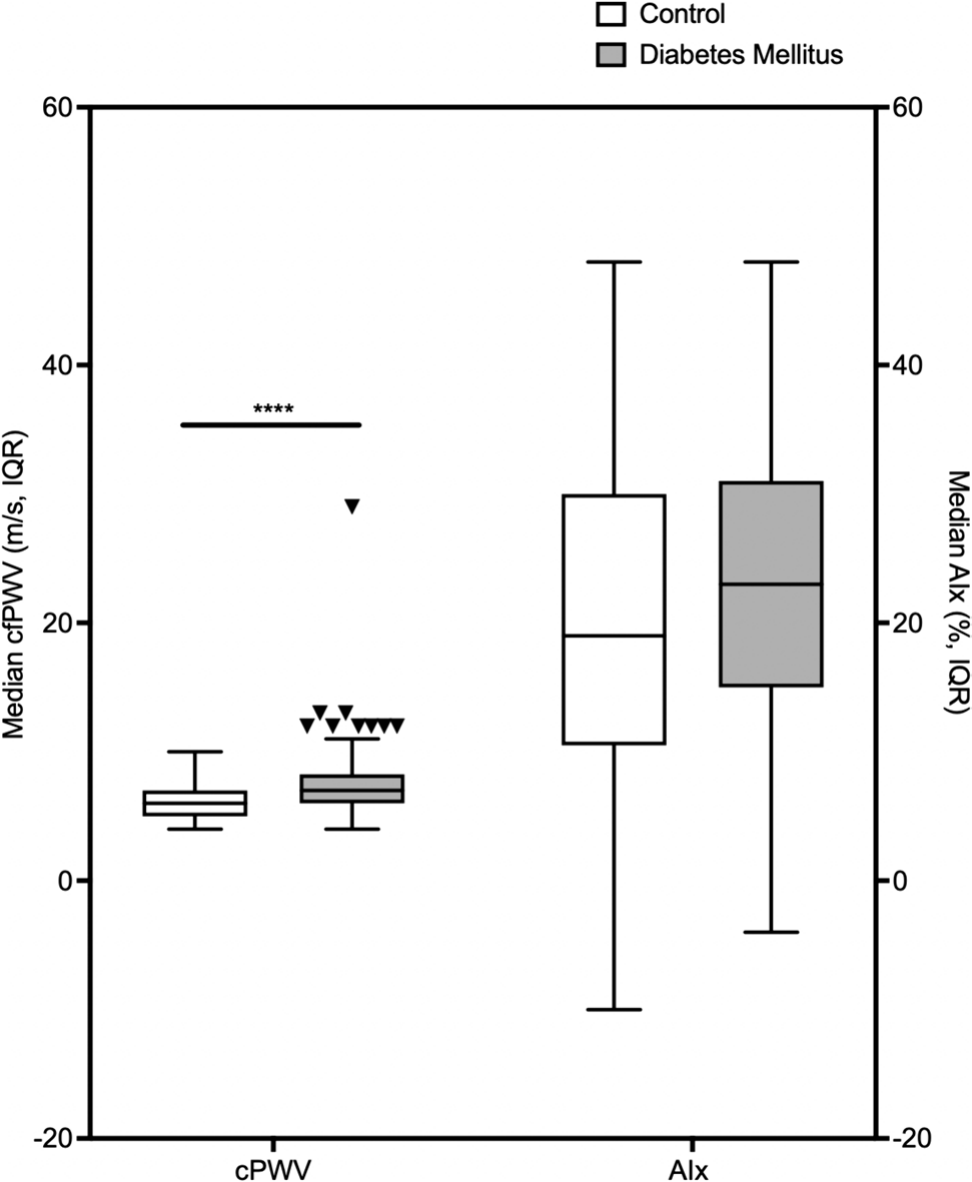
Comparison of median cfPWV and AIx with IQR values in controls and participants with diabetes mellitus. AIx, Augmentation index; cfPWV, carotid-femoral pulse wave velocity; IQR, interquartile range. ****p<0.0001

Figure 3 compares the median cfPWV and AIx in controls and participants with diabetes mellitus. The median cfPWV was higher in diabetes mellitus compared with controls (controls 5.80m/s, IQR 1.55 vs diabetes mellitus 7.00m/s, IQR 2.35, *p*<0.0001). The median AIx was higher in diabetes mellitus compared with controls but this did not reach statistical significance (controls 19.26%, IQR 19.94 vs diabetes mellitus 23.27%, IQR 14.94, *p*=0.28). There was also no correlation between cfPWV and AIx (r=0.13, *p*=0.15).

The time delay in retinal image initiation to acquisition between controls and participants with diabetes mellitus was calculated across four points of the cardiac cycle (0ms, 220ms, 300ms and 380ms). From the desired time of 0ms after the QRS, the actual time of photo acquisition was significantly before the QRS in participants with diabetes mellitus than controls (controls: -61.66ms, 95%CI -72.62- -51.04 vs diabetes mellitus: -39.40ms, 95%CI -50.34- -29.22, *p*=0.006). There was no statistically significant difference between the controls and participants with diabetes mellitus in actual times of photo acquisition compared to the desired times of 220ms (controls: 247.78ms, 95%CI 245.09-250.43 vs diabetes mellitus: 248.85ms, 95%CI 247.57-250.04, *p*=0.49), 300ms (controls: 305.85ms, 95%CI 304.07-308.11 vs diabetes mellitus: 305.85ms, 95%CI 304.73-307.00, *p*=1.00) and 380ms (controls: 386.90ms, 95%CI 385.01-389.20 vs diabetes mellitus: 386.90ms, 95%CI 385.67-388.18, *p*=1.00) after the QRS.

**Fig. 4:**
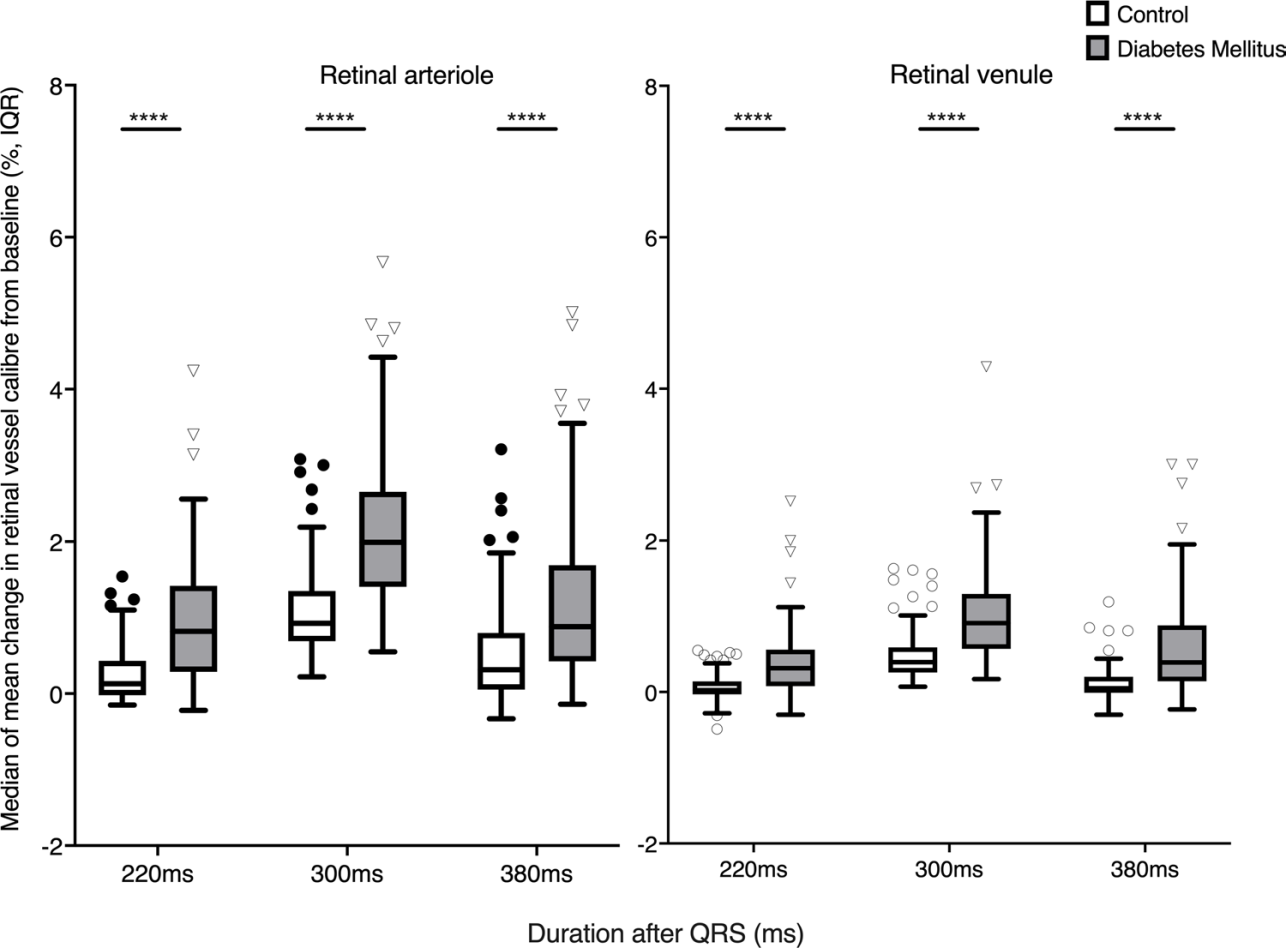
Median and IQR values comparing mean changes in retinal arteriolar and venular calibres at 220ms, 300ms and 380ms after the QRS from baseline (QRS) between controls and diabetes mellitus. IQR, interquartile range. ****p<0.0001

Figure 4 shows median changes in mean retinal arteriolar and venular calibres from baseline in controls and participants with diabetes mellitus**.** The mean changes in retinal arteriolar and venular calibres at all three time points after the QRS were significantly different from baseline (*p*<0.0001) in the controls and participants with diabetes mellitus. The highest arteriolar pulsatility and venular pulsatility occurred at 300ms after the QRS in both groups and both vessel types. A significantly higher median change in mean retinal arteriolar calibre from baseline was observed in participants with diabetes mellitus than controls at 220ms (controls: 0.13%, IQR 0.45 vs diabetes mellitus: 0.81%, IQR 1.12, *p*<0.0001), 300ms (controls: 0.92%, IQR 0.63 vs diabetes mellitus: 2.05%, IQR 1.25, *p*<0.0001) and 380ms (controls: 0.31%, IQR 0.77 vs diabetes mellitus: 0.88%, IQR 1.24, *p*<0.0001) after the QRS. A significantly higher median change in mean retinal venular calibre from baseline was also observed in participants with diabetes mellitus than controls at 220ms (controls: 0.02%, IQR 0.17 vs diabetes mellitus: 0.30%, IQR 0.48, *p*<0.0001), 300ms (controls: 0.40%, IQR 0.34 vs diabetes mellitus: 0.88%, IQR 0.71, *p*<0.0001) and 380ms (controls: 0.05%, IQR 0.22 vs diabetes mellitus: IQR 0.73, *p*<0.0001) after the QRS.

The correlation of cfPWV and AIx with arteriolar pulsatility and venular pulsatility in controls and participants with diabetes mellitus was determined. There was a weak positive linear correlation of cfPWV with arteriolar pulsatility (r=0.36, *p*<0.0001) and venular pulsatility (r=0.23, *p*=0.009). There was no correlation of AIx with cfPWV (r=0.11, *p*=0.21), arteriolar pulsatility (r=0.07, *p*=0.41) and venular pulsatility (r=0.01, *p*=0.87). There was a moderate linear correlation between arteriolar pulsatility and venular pulsatility (r=0.61, *p*<0.0001).

During the multiple linear stepwise regression analysis, arteriolar pulsatility was independently associated with diabetes mellitus (β=0.64, *p*=0.0005), cfPWV (β=0.09, *p*=0.01) and venular pulsatility (β=0.53, *p*<0.0001). The venular pulsatility was independently associated with arteriolar pulsatility only (β=0.58, *p*<0.0001). After adjusting for age, sex and heart rate, cfPWV was independently associated with arteriolar pulsatility (β=0.34, *p*=0.03). After adjusting for age, sex, heart rate, MAP, and BMI, AIx was independently associated with diabetes mellitus only (β=8.21, *p*=0.0002). Venular pulsatility and cfPWV were not associated with diabetes mellitus.

**Fig. 5:**
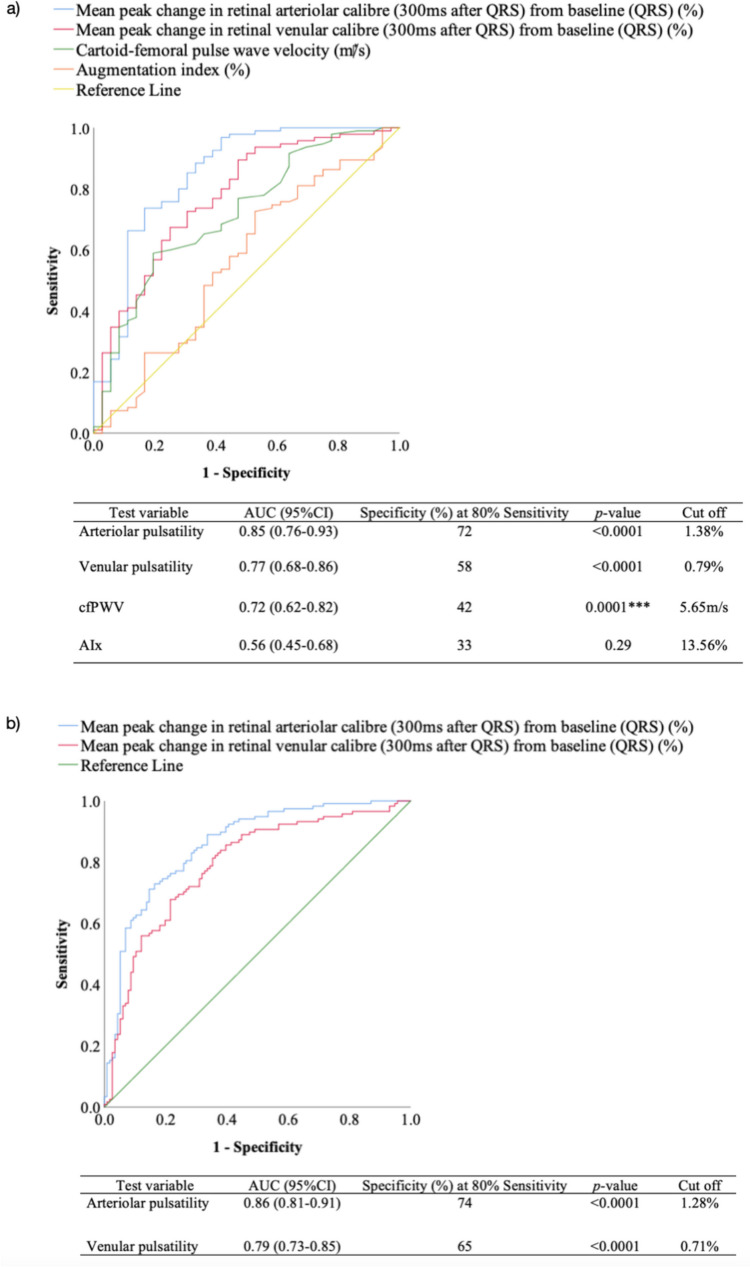
Receiver operating characteristic curve that compares the associations of diabetes mellitus with **a**) cfPWV, AIx, arteriolar pulsatility and venular pulsatility (n=95 participants with diabetes mellitus who completed both applanation tonometry and ECG-gated fundoscopy) and **b**) arteriolar pulsatility and venular pulsatility only (n=118 participants with diabetes mellitus who completed ECG-gated fundoscopy +/- applanation tonometry). cfPWV, carotid-femoral pulse wave velocity; AIx, Augmentation index. ***p<0.001

Figure 5 reflects the receiver operating characteristic curve that compared associations of diabetes mellitus with arterial applanation tonometry, arteriolar pulsatility and venular pulsatility. Both arterial applanation tonometry and the retinal examinations were performed on 36 controls and 95 participants with diabetes mellitus. Figure 5a shows that in these participants, the ability to discriminate between controls and participants with diabetes mellitus was good for arteriolar pulsatility, fair for venular pulsatility and cfPWV, and poor for AIx. The retinal examinations were performed on a total of 116 controls and 118 participants with diabetes mellitus, which included the subset of participants who had completed both arterial applanation tonometry and retinal examinations. Figure 5b shows that in these participants, the ability to discriminate between controls and participants with diabetes mellitus remained good for arteriolar pulsatility and fair for venular pulsatility.

The intra and inter-observer reliability of arteriolar pulsatility and venular pulsatility measurements was calculated in controls and participants with diabetes mellitus. The intra-observer reproducibility was high in retinal arterioles (ICC 0.87, 95%CI 0.71-0.95, *p*<0.0001) and venules (ICC 0.95, 95%CI 0.87-0.98, *p*<0.0001). The inter-observer reproducibility was also high in retinal arterioles (ICC 0.93, 95%CI 0.82-0.97, *p*<0.0001) and venules (ICC 0.97, 95%CI 0.92-0.99, *p*<0.0001). This demonstrated that there was good agreement of measurements by the same reviewer and between reviewers.

## Discussion

This study proposed an increased ECG-gated mean peak change in retinal vessel calibre from baseline in diabetes mellitus. The main objective was to assess the correlation of diabetes mellitus with systemic measures of arterial stiffness (arterial applanation tonometry) and mean peak changes in retinal vascular response from baseline. Another objective was to determine the precise timing of the cardiac cycle when minimal and maximal retinal vascular dilation occurred in controls and participants with diabetes mellitus. Retinal arteriolar pulsatility (and venular pulsatility) was more highly correlated with diabetes mellitus than cfPWV and AIx, and occurred 300ms after the QRS.

Previously [17], we demonstrated that in healthy individuals, the trough of the pressure waveform, representing minimum retinal arteriolar pulsatile flow, occurred at the end of diastole, on the QRS. The maximum retinal arteriolar pulsatile flow was observed to occur between 200-350ms after the QRS, corresponding with systole, but the exact timing was yet to be determined. With a larger sample size, and irrespective of diabetes status and vessel type, the present study determined that the maximum pulsatile flow occurred at 300ms after the QRS (late systole) and was in agreement with our previous study that the minimum pulsatile response occurred at approximately the QRS (end diastole).

Measurements of retinal calibre throughout the cardiac cycle have been explored previously, however, in small samples of only healthy subjects [18–20]. Hao *et al* [20] observed that retinal arterioles peaked before retinal venules in single and central retinal summary measurements, which was in contrast to our study that observed peak retinal arteriolar and venular diameter changes to occur at the same segment of the cardiac cycle (300ms after the QRS). This could be due to the time intervals of the cardiac cycle chosen for analysis in our study. Between 300ms and 380ms after the QRS, we do not know if there are variations in retinal venular diameter. Therefore, we cannot rule out the possibility that peak retinal venular diameter may in fact occur later, somewhere within this time range. The higher inter-photograph time variability when attempting to capture images at the QRS occurred because of the inherent time delay in our gating system (albeit the smallest delay reported in the literature) before images could be acquired after the QRS [12]. This meant image capture was based on an estimated time of the next heartbeat, which was particularly difficult to predict in younger participants with sinus arrhythmia. The time variability at 220ms, 300ms and 380ms after the QRS was small because predictions could be based on the same, known, heart cycle as when the acquire button was pushed.

Previous studies demonstrated that both retinal arterioles and venules were affected by cardiac cycle-induced pulsatile flow [18–20], which the present study has confirmed. The compositional and functional properties of the aorta and its main arteries could explain why retinal arterioles exhibit pulsatile flow. Large arteries closest to the heart serve as compliance vessels and have a high elastin to collagen ratio. This enables arterial expansion and accommodation of pulsatile pressure created from left ventricular ejection during systole, and recoil during diastole. The distensible characteristic in a high-pressure system allows the aorta to circulate blood to the retinal arterioles, dampening the majority, but not all, of the pulsatile flow. We did not expect pulsatility in retinal venules because the venous system is a low-pressure system consisting of capacitance vessels and is generally unaffected by pulsatile pressure from the cardiac cycle. However, a previous study [21] suggested that retinal venous pressure increased in diabetic retinopathy, resulting in a reduced perfusion pressure and increased transmural pressure that contribute to hypoxia and retinal oedema respectively. The majority of the participants with diabetes mellitus in our study had no retinopathy and whether retinal venous pulsatility is an earlier marker of subsequent pathological changes requires further investigation in future studies.

The higher arteriolar pulsatility in diabetes mellitus compared with controls may be a result of hyperglycaemia accelerating the process of arterial stiffening through oxidative stress and the production of advanced glycation end products that cross-link with collagen within the arterial wall [22]. The cross-linked collagen is resistant to degradation and favours the formation of additional collagen, thereby decreasing the elastin to collagen ratio in elastic vessels such as the aorta [23]. The excess collagen enhances vessel wall rigidity and decreases vessel wall compliance [24]. A stiffer aorta, in diabetes mellitus, reduces the aorta’s ability to dampen cardiac-generated pulsatile flow, which confers more pulsatile energy transfer to the retinal microcirculation [11].

However, whilst the present study observed the expected outcome of cfPWV being higher in participants with diabetes mellitus compared to controls, in accordance with previous studies [25–30], there was no correlation between cfPWV and diabetes mellitus after adjusting for confounding factors. This suggests that increased systemic arterial stiffness may not be associated with diabetes mellitus in our study population. On the other hand, we observed that AIx was higher in diabetes mellitus compared to controls after adjusting for confounding factors. The results for AIx are incongruous with a high intra-individual variability in AIx suggesting that measurements are unreliable [31] and questions its validity as a marker of arterial stiffness in the diabetes population. A generalised transfer function in predicting central aortic pressure from radial pressure remains controversial as it cannot account for all of the potential variables that may influence the estimated value [32]. There are also difficulties in creating accurate and reproducible aortic waveforms, which are required for precise calculations of AIx [7, 33]. Before AIx can be clinically useful, the aforementioned limitations must be overcome.

An alternative explanation to account for higher retinal arteriolar pulsatility in diabetes mellitus is that locally disturbed retinal autoregulatory responses in diabetes mellitus could be responsible for the inability of retinal vessels to respond appropriately to changes in systematic blood pressure. These autoregulatory changes in diabetes mellitus are associated with a higher risk of retinal arteriolar stiffness and have been observed even before the presence of clinically detectable diabetic retinopathy and systemic arterial stiffness [11]. The higher retinal arteriolar calibre change observed in diabetes mellitus due to a dysfunctional autoregulatory response could be responsible for accommodating this higher pulsatile flow. This may explain why we observed that higher arteriolar pulsatility was more strongly correlated with diabetes mellitus than a higher venular pulsatility, a higher cfPWV and a higher AIx, and the weak correlation observed in this study between retinal arterial pulsatility and cPWV.

Another reason why we did not observe a strong correlation between ECG-gated retinal vessel calibre and cfPWV could be in the procedure employed. The ECG-gated fundoscope may have been more accurate than arterial applanation tonometry in obtaining data. To be able to conclude this with certainty, the main limitation of this study, which is to establish the true degree of arterial stiffness to make direct comparisons between these two methods, needs to be overcome. A precise depiction of arterial stiffness can be achieved by non-invasive methods, including velocity-encoded MRI as it is validated against catheterisation (invasive gold standard) [5], and echocardiography that permits direct visual access to the arterial wall and blood flow measurements in real-time [4]. A large population-based cohort study would also be required that will improve the external validity. The direction of causality between arteriolar pulsatility and diabetes mellitus could also not be established due to the cross-sectional design of this study. Other limitations of this study include lack of access to fasting blood sugar information for many individuals, as well as limited number of patients with diabetic retinopathy which did not permit determining associations between retinal arteriolar pulsatility and the degree of diabetic retinopathy. This could be considered in future studies for the purposes of risk stratification.

Nevertheless, this study had some major strengths including being the first study to determine the correlation of arterial applanation tonometry and pulsatile changes in retinal vessel calibre with diabetes mellitus. We reported high intra- and interobserver correlations of arteriolar pulsatility and venular pulsatility, using randomly selected slice points, in the same retinal vessels, demonstrating that this method is reliable and repeatable.

In conclusion, higher arteriolar pulsatility has a stronger association with diabetes mellitus than arterial applanation tonometry. This finding is clinically useful in better understanding retinal pathophysiology from the circulation perspective. However, further research is still required to fully elucidate the screening potential of arteriolar pulsatility as an early risk marker for arterial stiffness in diabetes mellitus.

## Data Availability

The raw quantitative dataset used to support the findings of this study are deidentified participant data and are available from the corresponding author on reasonable request. Please contact Dr. Anchal Lal: alal2824@alumni.sydney.edu.au if interested.
